# Intronic Enhancer Is Essential for *Nr5a1* Expression in the Pituitary Gonadotrope and for Postnatal Development of Male Reproductive Organs in a Mouse Model

**DOI:** 10.3390/ijms24010192

**Published:** 2022-12-22

**Authors:** Yuichi Shima, Kanako Miyabayashi, Takami Mori, Koji Ono, Mizuki Kajimoto, Hae Lim Cho, Hitomi Tsuchida, Yoshihisa Uenoyama, Hiroko Tsukamura, Kentaro Suzuki, Man Ho Choi, Kazunori Toida

**Affiliations:** 1 Division of Microscopic and Developmental Anatomy, Department of Anatomy, Kurume University School of Medicine, 67 Asahi-machi, Kurume 830-0011, Fukuoka, Japan; 2 Department of Anatomy, Kawasaki Medical School, 577 Matsushima, Kurashiki 701-0192, Okayama, Japan; 3 Department of Medical Technology, Faculty of Health Science and Technology, Kawasaki University of Medical Welfare, 288 Matsushima, Kurashiki 701-0193, Okayama, Japan; 4 School of Medical Technology, Faculty of Health and Medical Care, Saitama Medical University, 1397-1 Yamane, Hidaka 350-1241, Saitama, Japan; 5 Pharmacokinetics and Bioanalysis Center, Shin Nippon Biomedical Laboratories, Ltd. (SNBL), 16-1 Minamiakasaka, Kainan-shi 642-0017, Wakayama, Japan; 6 Center for Advanced Biomolecular Recognition, Korea Institute of Science and Technology, 5 Hwarang-ro 14-gil, Seoul 02792, Republic of Korea; 7 Laboratory of Animal Reproduction, Graduate School of Bioagricultural Sciences, Nagoya University, Nagoya 464-8601, Aichi, Japan; 8 Faculty of Life and Environmental Sciences, University of Yamanashi, 4-4-37, Takeda, Kofu 400-8510, Yamanashi, Japan

**Keywords:** NR5A1, enhancer, pituitary gonadotrope, luteinizing hormone, follicle-stimulating hormone

## Abstract

Nuclear receptor subfamily 5 group A member 1 (NR5A1) is expressed in the pituitary gonadotrope and regulates their differentiation. Although several regulatory regions were implicated in *Nr5a1* gene expression in the pituitary gland, none of these regions have been verified using mouse models. Furthermore, the molecular functions of NR5A1 in the pituitary gonadotrope have not been fully elucidated. In the present study, we generated mice lacking the pituitary enhancer located in the 6th intron of the *Nr5a1* gene. These mice showed pituitary gland-specific disappearance of NR5A1, confirming the functional importance of the enhancer. Enhancer-deleted male mice demonstrated no defects at fetal stages. Meanwhile, androgen production decreased markedly in adult, and postnatal development of reproductive organs, such as the seminal vesicle, prostate, and penis was severely impaired. We further performed transcriptomic analyses of the whole pituitary gland of the enhancer-deleted mice and controls, as well as gonadotropes isolated from Ad4BP-BAC-EGFP mice. These analyses identified several genes showing gonadotrope-specific, NR5A1-dependent expressions, such as *Spp1*, *Tgfbr3l*, *Grem1*, and *Nr0b2*. These factors are thought to function downstream of NR5A1 and play important roles in reproductive organ development through regulation of pituitary gonadotrope functions.

## 1. Introduction

Sex hormone secretion from the gonads is controlled by pituitary gonadotropins, the production and secretion of which is controlled by the hypothalamic gonadotropin-releasing hormone (GnRH). This hierarchical sex hormone production control mechanism is called the hypothalamic-pituitary-gonadal (HPG) axis. Pituitary gonadotropins include luteinizing hormone (LH) and follicle-stimulating hormone (FSH), and they are composed of common α subunit and unique β subunits, LHβ and FSHβ, respectively. LH stimulates testicular Leydig cells to produce androgens, whereas FSH stimulates Sertoli cells to support spermatogenesis. Male LHβ and LH receptor knockout mice show normal masculinization at fetal periods but severely impaired postnatal reproductive organ development, indicating the physiological importance of LH in male reproductive function [1,2]. FSHβ knockout mice show milder phenotypes than LHβ/LH receptor knockout mice, indicating a minor role of FSH in male reproductive function [3].

One of the most important factors for HPG axis formation is the nuclear receptor subfamily 5 group A member 1 (NR5A1, also known as Ad4-binding protein (Ad4BP) or Steroidogenic Factor-1 (SF-1)). Although this factor is not expressed in the hypothalamic GnRH neurons, it is expressed not only in pituitary gonadotropes but also in the ventromedial hypothalamic nucleus, adrenal cortex, Sertoli and Leydig cells of the testis, and granulosa and theca cells of the ovaries [4,5]. In mice, systemic *Nr5a1* gene disruption resulted in structural and functional abnormalities in all these tissues, indicating the pivotal roles of NR5A1 in each tissue [6]. However, because systemic *Nr5a1* knockout mice die in the neonatal period due to adrenal insufficiency, tissue-specific functions of NR5A1 are not well understood. In order to clarify the NR5A1 function in the pituitary, pituitary-specific conditional *Nr5a1* knockout mice have been generated using the αGSU-Cre lineage [7,8]. In these mice, the expression of *Lhb* and *Fshb* is abrogated, indicating that NR5A1 plays an important role in the functional differentiation of the pituitary gonadotropes.

As *Nr5a1* is expressed in multiple tissues, several research groups including our group have performed transgenic mouse assays to identify tissue-specific regulatory regions. These analyses have identified several enhancers such as the fetal adrenal enhancer (FAdE) [9], ventromedial hypothalamus enhancer (VE) [10], pituitary enhancer (PE) [11], and fetal Leydig enhancer (FLE) [12] in the *Nr5a1* gene locus. Although these enhancers have been identified by generating transgenic mice, functional importance of these enhancers has not been directly verified by genome deletion. We recently used genome editing to generate mice with FLE deletion, which showed fetal Leydig cell (FLC)-specific NR5A1 deficiency and severe defects in male reproductive organs from fetal stages, clearly demonstrating the indispensable role of FLE in FLC-specific *Nr5a1* expression [13]. Based on this result, in this study, we generated a mouse line lacking the PE of *Nr5a1* and confirmed that PE plays an essential role in pituitary-specific *Nr5a1* expression.

NR5A1 begins to express in the anterior pituitary at E13.5–E14.5, and analysis of *Nr5a1*-disrupted mice suggested that NR5A1 regulates gonadotropin production in the pituitary gonadotrope [14]. In addition, the results of in vitro analysis suggested that *Lhb* and *Cga* expression was directly controlled by NR5A1 [15,16]. On the other hand, the expression of LHβ and FSHβ was reduced but not completely lost in the pituitary gland–specific *Nr5a1* knockout mice [7,8]. Furthermore, the expression of LHβ and FSHβ is induced by GnRH stimulation in *Nr5a1* gene knockout mice [14], suggesting that NR5A1 is not essential for LHβ and FSHβ expression. Considering these results together, it is conceivable that there are other downstream genes that are directly regulated by NR5A1 in pituitary gonadotropes. In this study, we identified several candidate NR5A1 downstream genes. Some of these genes has not been previously linked to the pituitary gonadotrope functions and might be worth for further analyses in future studies.

## 2. Results

### 2.1. Deletion of Gonadotrope-Specific PE of Nr5a1

**Figure 1:**
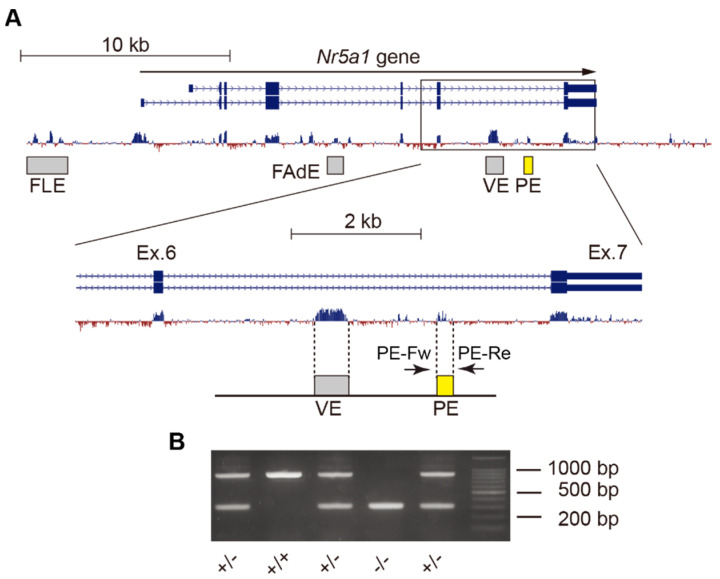
Deletion of the pituitary enhancer (PE) of *Nr5a1* by genome editing. (**A**) *Nr5a1* comprises seven exons. Several tissue-specific enhancers exist in the intronic and upstream regions of *Nr5a1*, such as the fetal Leydig enhancer (FLE), fetal adrenal enhancer (FAdE), ventromedial hypothalamic nucleus enhancer (VE), and PE. (**B**) The PE (yellow filled box) in the sixth intron of the gene was deleted by genome editing, and genotyping PCR with the primers PE-Fw and PE-Re confirmed that the region was successfully removed from the genome.

To confirm the functional significance of the PE, we adopted CRISPR/Cas9 genome editing to delete the PE region (Figure 1A). Genotyping PCR and direct sequencing confirmed that the PE region was successfully deleted from the mouse genome (Figure 1B and Figure S1). Both male and female PE^+/−^ (heterozygous deletion of the enhancer) mice were fertile, but PE^−/−^ (homozygous deletion of the enhancer) mice were infertile in both males and females.

### 2.2. Normal Masculinization in Fetal Stages

#### 2.2.1. Testis and Accessory Reproductive Organs

**Figure 2:**
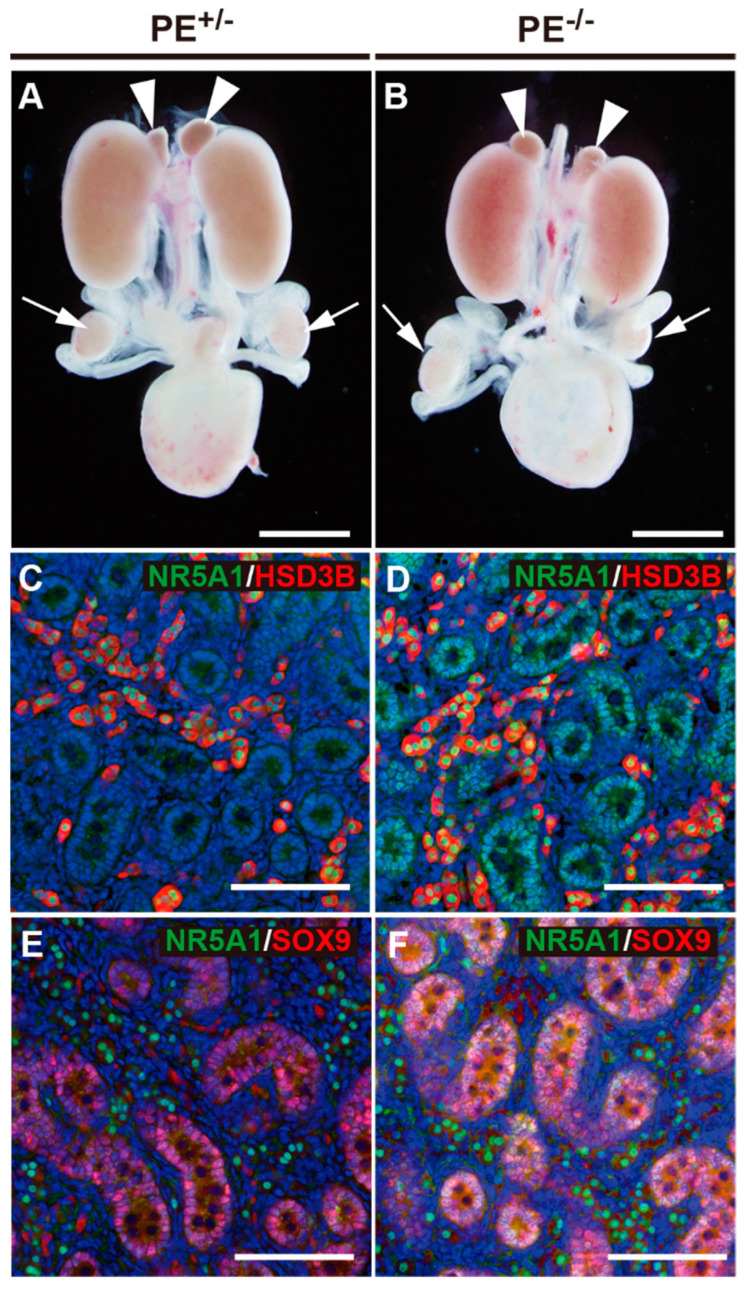
Fetal masculinization was not affected in PE^−/−^ mice. (**A**,**B**) Macroscopic observation of the urogenital systems in the control mice (**A**) and PE^−/−^ mice (**B**). Arrowheads indicate adrenal glands, whereas arrows indicate testes. (**C**,**D**) Double staining of the testis with antibodies for NR5A1 (green) and HSD3B (red). (**E**,**F**) Double staining of the testis with NR5A1 (green) and SOX9 (red) antibodies. Scale bar: 2 mm in (**A**,**B**), 100 μm in (**C**–**F**).

Because PE deficiency was expected to abolish pituitary Nr5a1 expression and reduce the function of the pituitary gonadotropes, we examined the phenotype of PE^−/−^ male mice in comparison with that of control (PE^+/−^) mice to analyze the effects of enhancer deficiency. Fetal PE^−/−^ male mice (embryonic day 18.5; E18.5) showed normal descendance and size of the testis relative to those in the control mice (arrows in Figure 2A,B). In addition, vas deferens development and adrenal gland size (arrowheads in Figure 2A,B) were consistent between groups, confirming that the effect of enhancer deficiency was limited to the pituitary gonadotrope. Immunostaining revealed that NR5A1 was strongly expressed in Leydig cells in the interstitium of the testis and weakly expressed in Sertoli cells in the seminiferous tubules of the testis in PE^−/−^ mice, showing no clear differences from expression patterns in control mice. Accordingly, no abnormality was observed in HSD3B expression in Leydig cells or SOX9 expression in Sertoli cells (Figure 2C–F).

#### 2.2.2. Steroids in Fetal Testes

The intratesticular concentration of steroid hormones was evaluated by GC-MS. Testosterone and androstenedione levels were slightly higher in PE^−/−^ mice than in the control mice, but the difference was not significant (Table 1). Levels of other steroids showed no significant differences in concentration between control and PE^−/−^ testis. There was also no significant difference between control and PE^−/−^ testes in the metabolic ratio of enzymatic reactions required for androgen synthesis (Supplemental Table S1).

### 2.3. Impaired Development of Reproductive Organs at Adult Stages

#### 2.3.1. Testis

**Figure 3:**
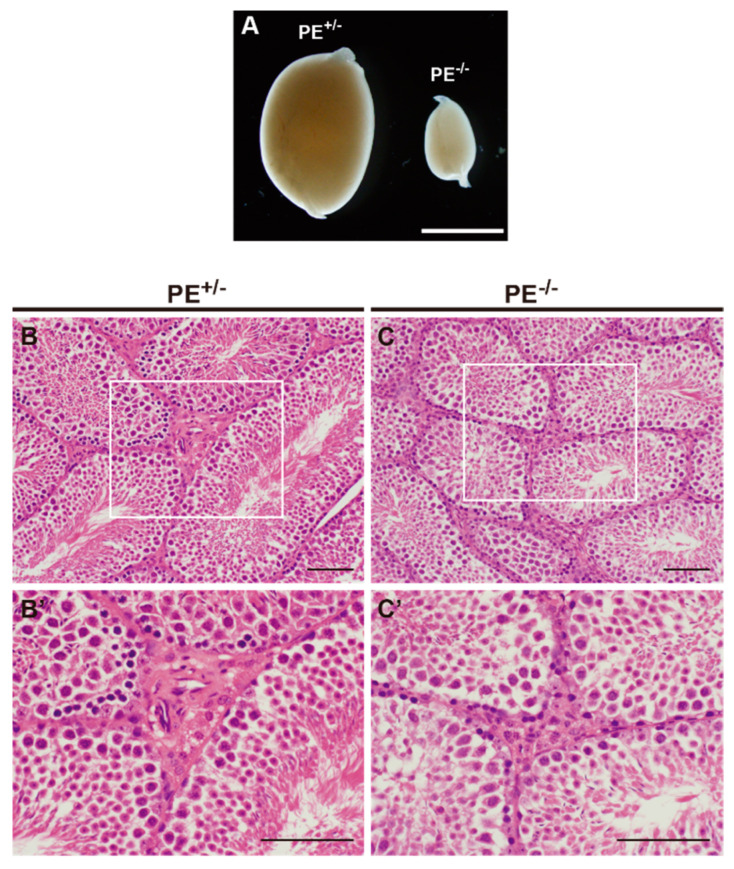
Severely impaired testicular architecture in PE^−/−^ adult mice. (**A**) Macroscopic view of the testes collected from control and PE^−/−^ mice. (**B**,**C**) Low magnification view of the HE stained section of testes collected from control (**B**) and PE^−/−^ mice (**C**). (**B**’,**C**’) Magnified view of the areas enclosed by open rectangles in (**B**,**C**). Scale bar: 2 mm in (**A**), 100 µm in (**B**–**C**’).

The testes of adult male PE^−/−^ mice were significantly smaller than those of control mice (Figure 3A), whereas the size of the adrenal gland was unaffected (Figure S2). Hematoxylin and eosin (HE) staining of testis sections revealed that the diameter of the seminiferous tubules was clearly reduced in PE^−/−^ mice relative to that in controls, and few mature spermatozoa were found within the seminiferous tubules (Figure 3B,C). The area of the testicular interstitium was also narrower in PE^−/−^ than in control mice, and lipid droplets within the interstitial Leydig cells were reduced (Figure 3B’,C’).

Female PE^−/−^ mice exhibited smaller ovaries than control mice (Figure S3A). When tissue sections were prepared and analyzed, the PE^−/−^ mice did not present a large number of corpora lutea compared to those found in the ovaries of control mice (Figure S3B,B’,C). This was thought to be because ovulation did not occur due to decreased LH secretion from the pituitary. In addition, many traces of closed follicles were observed in the ovaries (arrowheads in Figure S3C’), and it was speculated that the follicles could not be maintained due to the decrease in estrogen.

#### 2.3.2. Seminal Vesicles, Prostate Gland, and Penis

**Figure 4:**
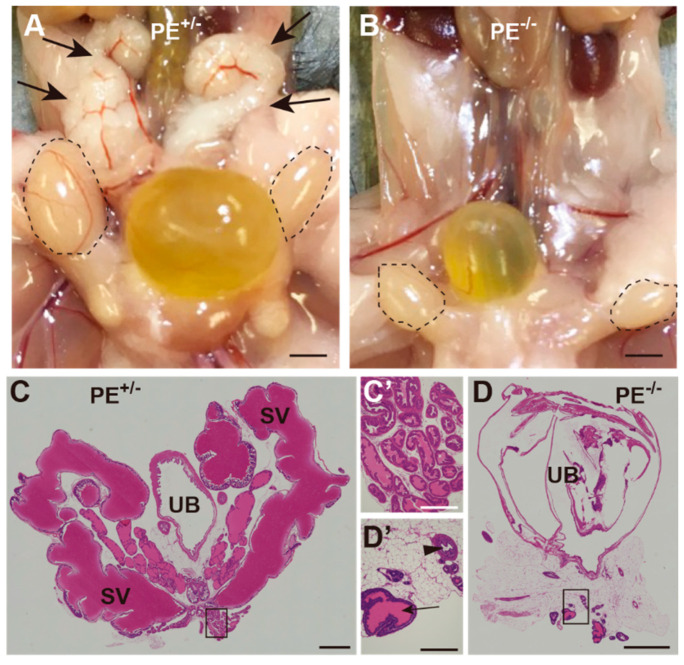
Hypoplastic development of reproductive organs in PE^−/−^ male mice. (**A**,**B**) Macroscopic views of the lower abdominal organs in control (**A**) and PE^−/−^ (**B**) mice. Testes are encircled by broken lines. Arrows in (**A**) indicate seminal vesicles, which were not observed in PE^−/−^ mice (**B**). (**C**,**D**) HE-stained sections of the urinary bladder (UB) and seminal vesicles (SV) in control (**C**) and PE^−/−^ (**D**) mice. (**C**’,**D**’) Magnified view of the areas enclosed by open rectangles in (**C**,**D**). An arrow in (**D**’) indicates a rudimentary tissue of the prostate gland. An arrowhead in (**D**’) indicates the urethra. Scale bar: 2 mm in (**A**–**D**); 100 µm in (**C**’,**D**’).

**Figure 5:**
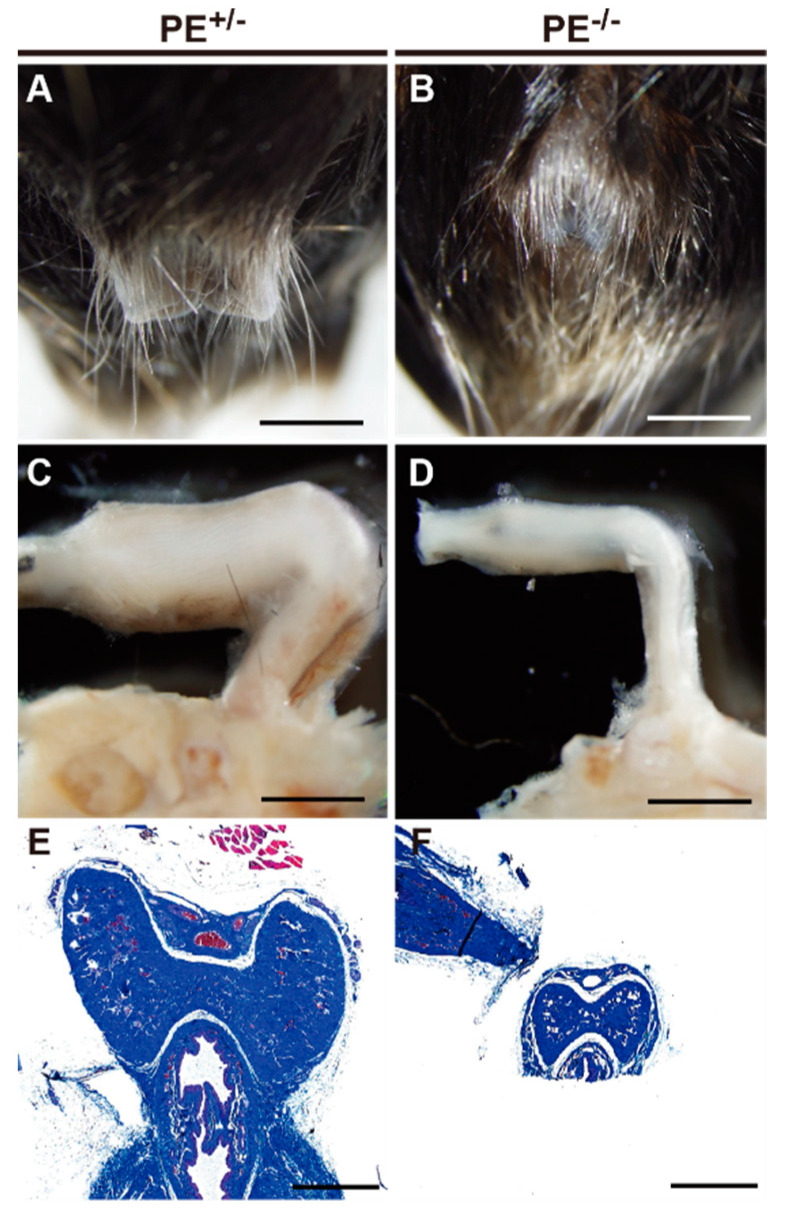
Hypoplastic penis in PE^−/−^ male mice. (**A**,**B**) Macroscopic view of the penis of control (**A**) and PE^−/−^ (**B**) mice. (**C**,**D**) Penile foreskin was removed, and penes were compared between control (**C**) and PE^−/−^ (**D**) mice. (**E**,**F**) Masson-trichrome staining of the penile sections of control (**E**) and PE^−/−^ (**F**) mice. Scale bar: 2 mm in (**A**–**D**); 500 µm in (**E**,**F**).

Macroscopic observation of accessory reproductive organs showed that seminal vesicles were not apparent in PE^−/−^ male mice (Figure 4A,B). HE staining revealed that most of the seminal vesicles and prostate had been replaced with adipose tissue, and only a few traces of the prostate glands were identified (Figure 4C–D’). The external genitalia were also clearly smaller in appearance in the PE^−/−^ mice than in the controls (Figure 5A,B), and the size of the penis was also reduced (Figure 5C,D). Masson-trichrome staining clearly showed poor development of the penis in PE^−/−^ males (Figure 5E,F).

#### 2.3.3. Steroid Levels in Adult Testes

GC-MS revealed the presence of several steroids in adult testes that were not detected in the fetal testes, such as 3α-androstane-3α,17β-diol, 7α-hydroxyandrostenedione, dihydrotestosterone, 6β-hydroxyandrostenedione, 6β-hydroxytestosterone, 16α-hydroxytestosterone, 16α-hydroxyandrostenedione, 17α-hydroxypregnenolone, tetrahydrodeoxycorticosterone, allo-tetrahydrodeoxycorticosterone, and corticosteron. Among detected steroids, dehydroepiandrosterone, androstenediol, 7α-hydroxyandrostenedione, androstenedione, and testosterone were significantly lower in the testes of PE^−/−^ mice than in those of control mice (Table 2). From these results, it was speculated that the activities of 17α-hydroxylase/17,20-lyase, 3β-HSD, and 17β-HSD, enzymes involved in the synthesis of testosterone, were globally reduced. Indeed, comparison of metabolic ratios between control testes and PE^−/−^ testes suggested that activities of enzymes, such as 21-hydroxylase, 17,20-lyase, 3β-HSD, 17β-HSD, 17α-HSD, 5α-reductase, and 3α-HSD were significantly decreased in PE^−/−^ testes (Supplemental Table S2).

#### 2.3.4. Quantitative Reverse Transcription (qRT)-PCR and Gonadotropin Immunostaining

**Figure 6:**
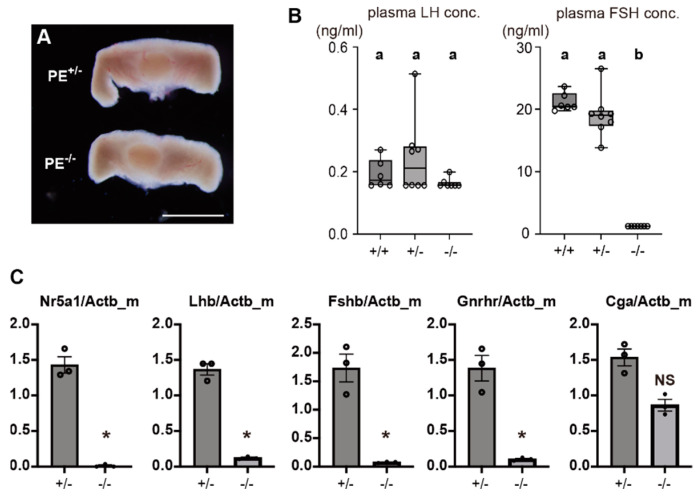
Changes in pituitary gonadotrope marker genes in PE^−/−^ mice. (**A**) Macroscopic view of the pituitary glands of control and PE^−/−^ mice. Scale bar: 1 mm (**B**) Plasma concentrations of LH and FSH in control (PE^+/+^ and PE^+/−^) and PE^−/−^ mice. Differences were evaluated by one-way ANOVA followed by Tukey’s post hoc test at a significance level of *p* < 0.05. a and b: significant difference between different characters. (**C**) Relative expression of pituitary gonadotrope marker genes in control and PE^−/−^ mice, as evaluated by qRT-PCR. Y-axis represents gene expression relative to that of *Actb*. Statistical significance between two experimental groups was evaluated by unpaired *t*-test. * significant difference (*p* < 0.05), NS: not significant.

The pituitary glands of PE^−/−^ mice showed no apparent size difference compared to those of the control mice (Figure 6A). Plasma LH levels tended to be lower in PE^−/−^ mice than in control mice, but no significant difference was detected because LH concentrations were generally low in control mice. FSH concentration was significantly lower in PE^−/−^ mice than in the control group (Figure 6B).

RNA was extracted from the pituitary gland, and the expression of marker genes of gonadotropin-producing cells was analyzed by qRT-PCR. In male and female PE^−/−^ mice, *Nr5a1* expression was almost completely absent, while that of *Lhb*, *Fshb*, and *Gnrhr* was detectable but significantly reduced relative to control values. *Cga* expression in male PE^−/−^ mice was reduced to about half that of control mice; expression in females was reduced to about 70% of the control value (Figure 6C and Figure S4).

**Figure 7:**
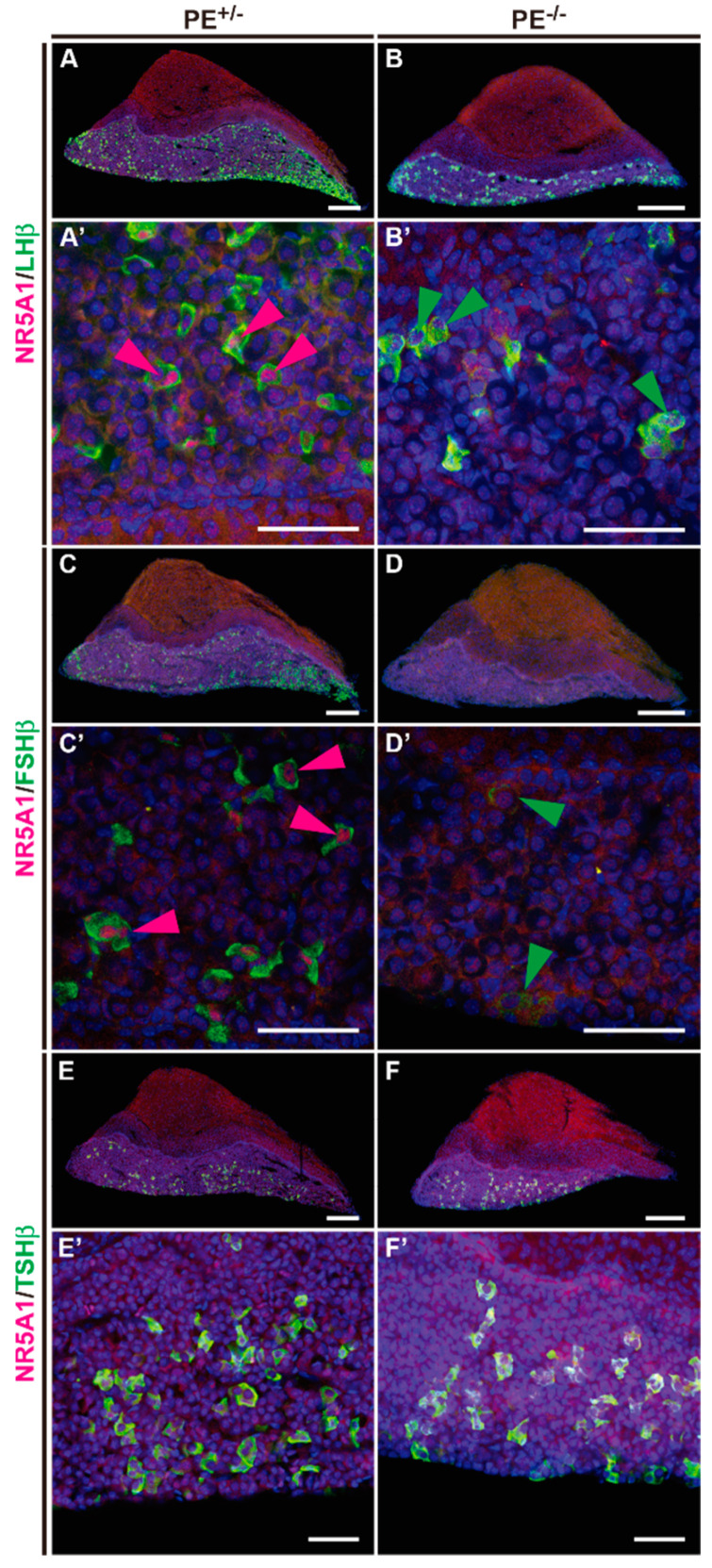
Levels of LHβ and FSHβ decreased in the PE^−/−^ mouse pituitary; TSHβ levels did not change. (**A**–**B**’) Immunostaining of pituitary sections using antibodies for NR5A1 (red) and LHβ (green). (**C**–**D**’) Immunostaining of pituitary sections using antibodies for NR5A1 (red) and FSHβ (green). (**E**–**F**’) Immunostaining of pituitary sections using antibodies for NR5A1 (red) and TSHβ (green). Scale bars: 200 μm in (**A**–**F**), 50 μm in (**A**’–**F**’).

Expression of LHβ, FSHβ, and thyroid stimulating hormone β (TSHβ) was examined by immunostaining. A considerable number of LHβ-expressing cells were present in the pituitary gland of PE^−/−^ mice (Figure 7A,B). These LHβ-expressing cells did not show nuclear NR5A1 expression, suggesting that NR5A1 was not essential for LHβ expression (Figure 7A’,B’). The number of FSHβ-expressing cells was dramatically reduced in the PE^−/−^ group relative to that in the controls (Figure 7C,D). However, cells weakly expressing FSHβ were still observed in the pituitary of PE^−/−^ mice, suggesting that NR5A1 influenced FSHβ expression (Figure 7C’,D’). NR5A1 was not expressed in TSHβ-expressing cells, and no obvious abnormalities in TSHβ expression were observed in PE^−/−^ mice relative to control expression (Figure 7E–F’).

### 2.4. Transcriptome Analyses of Pituitaries and Isolated Gonadotropes

**Figure 8:**
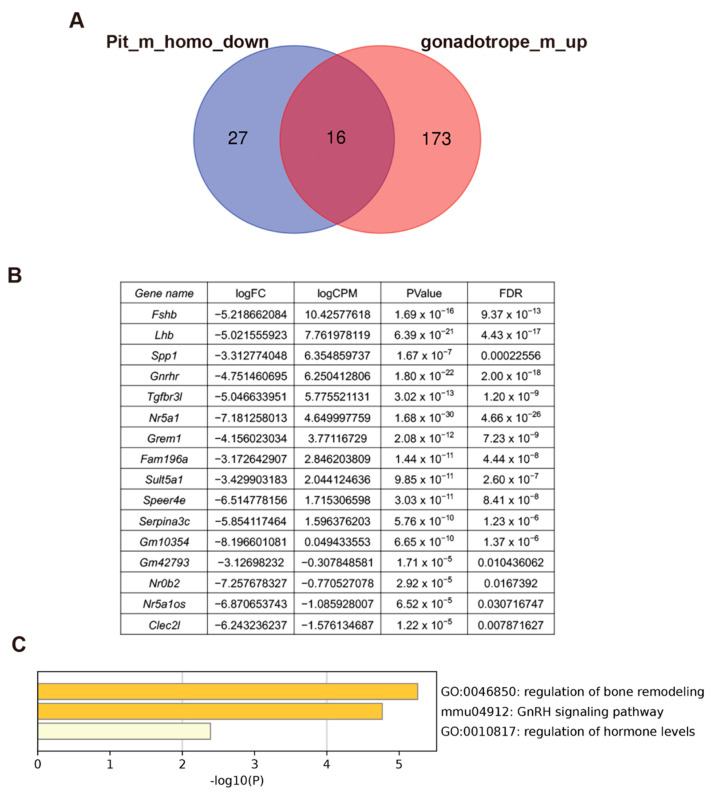
Transcriptomic analyses of the whole pituitary gland and isolated gonadotropes in males. (**A**) Venn diagram showing an overlap between genes downregulated in PE^−/−^ pituitary (Pit_m_homo_down) relative to the control and those showing higher expression in the gonadotropes than in the whole pituitary (gonadotrope_m_up). (**B**) List of the 16 overlapping genes of the two gene sets shown in (**A**); differential expression of 16 genes between control and PE^−/−^ pituitary glands. (**C**) GO terms enriched in the 16 genes.

Because the expression of *Lhb* and *Fshb* was not completely lost in PE^−/−^ mice, we searched for other downstream genes directly regulated by NR5A1. We first analyzed the transcriptome of the entire pituitary gland and extracted 43 genes with reduced pituitary expression in PE^−/−^ mice compared to that in controls (pit_m_homo_down; Figure 8A). Thereafter, we analyzed the transcriptome of the isolated gonadotropes and compared it with that of the whole pituitary, identifying 189 highly expressed genes in the gonadotropes relative to whole pituitary expression (gonadotrope_m_up; Figure 8A). In this process, we noticed that one of the isolated gonadotrope samples (gonadotrope_m1) showed a distinct gene expression pattern from the other three (Figure S5) and excluded this sample from the analysis. By comparing the pit_m_homo_down and gonadotrope_m_up gene sets, we identified 16 genes with gonadotrope-specific, NR5A1-dependent expression (Figure 8B). Gene ontology (GO) analysis of these genes highlighted “regulation of bone remodeling,” “GnRH signaling pathway,” and “regulation of hormone levels” as highly enriched GO terms (Figure 8C). We performed the same analyses in female samples and identified nine genes enriched in “gonad development” and “neuroactive ligand-receptor interaction” (Figures S6 and S7).

## 3. Discussion

### 3.1. Functional Importance of the PE in Nr5a1 Gene Regulation

*Nr5a1* contains multiple internal and upstream regulatory regions (enhancers). Although these enhancers have been identified by generating transgenic mice [9,10,11,12], their functional importance has not been strictly defined. In our previous study, the PE of *Nr5a1* was identified in the sixth intron [11]. In this study, we demonstrated that deletion of this PE leads to cell-specific and complete NR5A1 deficiency. In a recent study by another group, the ATAC-sequence of pituitary gonadotrope–derived cell lines suggested that regions other than the PE (the FLE and a small region in the fourth intron) are also implicated in *Nr5a1* expression in the pituitary gonadotropes [17]. The involvement of these regions (especially functionally undefined region in the fourth intron) in pituitary-specific NR5A1 expression should be carefully investigated in future studies.

### 3.2. Dependence of Fetal and Adult Leydig Cells on Pituitary Gonadotropins

The phenotype of PE-deficient mice was essentially the same as that previously reported in mice with pituitary-specific *Nr5a1* gene disruption [7,8]. That is, adult male mice were infertile due to insufficient formation of reproductive organs and reduced production of androgens. In females, ovulation did not occur, and the corpus luteum did not form, causing infertility. Furthermore, no defects were observed in the masculinization of fetal PE^−/−^ male mice. These data suggest that fetal masculinization proceeds in a pituitary-independent manner. Previous studies have shown that even when LHβ or LH receptors are deleted, fetal masculinization proceeds normally, but the production of androgens after birth declines and puberty does not occur, leading to defective spermatogenesis and hypoplastic male reproductive organs [1,2]. Another example is the *Kiss1* knockout mouse. In these mice, kisspeptin-induced GnRH production is absent and blood LH levels are decreased, but fetal androgen production is unaffected, whereas postnatal androgen production is markedly reduced [18]. These results were explained by the pituitary gland-independent development of Leydig cells in fetal testes, and LH-dependent Leydig cell development in postnatal testes [19]. To support this notion, our previous study showed that fetal Leydig cell–specific LH receptor knockout mice exhibited normal reproductive organs at the fetal stage [20].

The production of male hormones has been reported to be triphasic, comprising fetal, neonatal, and adolescent periods [21]. Fetal Leydig cells are responsible for the production of male hormones during the fetal period, and adult Leydig cells after puberty. In addition, transient HPG axis activation during the neonatal period is known to produce male sex hormones through a process called mini-puberty. Recent studies have focused on the influence of mini-puberty on spermatogenesis and male reproductive function at adult stages [22]. PE-deficient mice may represent a useful tool to clarify the physiological significance of mini-puberty.

### 3.3. Role of NR5A1 in the Pituitary Gonadotrope

Analysis of *Nr5a1*-disrupted mice suggested that NR5A1 is important for the functional differentiation of pituitary gonadotropes. Moreover, from the results of in vitro analysis, *Lhb* and *Cga* expression been reported to be directly controlled by NR5A1 [15,16]. However, in both this study and the previous works [7,8], the expression of LHβ and FSHβ was reduced but not completely lost in the pituitary gland–specific *Nr5a1* knockout mice. Furthermore, the expression of LHβ and FSHβ is also induced by GnRH stimulation in *Nr5a1* gene knockout mice [14], suggesting that NR5A1 is not essential for LHβ and FSHβ expression. These results suggested that there may be other downstream genes that are directly regulated by NR5A1 in pituitary gonadotropes. These genes might be related to GnRH responsiveness, signal transduction downstream of the GnRH receptor, or gonadotropin secretion, and several studies have been performed to identify such genes.

### 3.4. Candidate NR5A1 Downstream Genes

From the results of transcriptome analyses, 16 NR5A1-dependent genes with high expression in isolated gonadotropes were identified. GO analyses of these 16 genes identified *Fshb*, *Spp1*, and *Grem1* as related to “regulation of bone remodeling.” The *Spp1* gene encodes secreted phosphoprotein 1, or osteopontin, which shows gonadotrope-specific pituitary expression and regulates the interaction between gonadotropes and extracellular matrices [23]. Interestingly, osteopontin shows higher expression in male than in female gonadotropes. In agreement with this, our results showed that *Spp1* was highly expressed in the male gonadotrope but not in the female gonadotrope. *Grem1* encodes Gremlin1, an antagonist of bone morphogenetic protein. In a previous study, *Grem2*-null mice showed irregular estrous cycles and subfertility [24]. Although *Grem2* is not expressed in the pituitary gland, these previous data suggested that Grem2 plays an important role in HPG axis regulation and reproductive function in females. Our study expands on this to suggest that Grem1 is a novel regulator of gonadotrope function in males. *Tgfbr3l,* which encodes transforming growth factor β receptor III-like protein and plays essential roles in the transduction of inhibin B signaling to the pituitary gonadotrope, was also included in the gene set. Recently generated *Tgfbr3l* gene-disrupted female mice showed increased FSH production and follicle development relative to controls, and double knockout of *Tgfbr3l* and betaglycan resulted in female infertility [25], indicating an important role of this factor in female reproductive function. Another recent study identified an NR5A1 binding sequence in the proximal promoter of the human and murine *Tgfbr3l* homologs, and in vitro analyses suggested that NR5A1 directly induces gonadotrope-specific *Tgfbr3l* gene expression [26]. Our study supported this finding and strongly suggested that NR5A1 directly regulates *Tgfbr3l* gene expression in vivo. The *Tgfbr3l* gene also shows gonadotrope-specific and NR5A1-dependent expression in males. However, its role in male reproductive function has not been clarified. *Nr0b2* encodes a small heterodimer partner (SHP), a factor known to regulate bile acid homeostasis [27]. Recent studies have focused on its function in the testes [27], but the physiological function of SHP in the pituitary gonadotrope has not been investigated so far. Another *Nr0b* family gene, *Nr0b1*, showed gonadotrope-specific and NR5A1-dependent expression in females. This gene encodes dosage-sensitive sex reversal, adrenal hypoplasia congenita critical region, on chromosome X, gene 1 (DAX-1) [27]. Previous studies have shown that DAX-1 expression overlaps with that of NR5A1 in various tissues, including pituitary gonadotropes [28]. Although several previous in vitro studies have suggested that DAX-1 is directly regulated by NR5A1 [29,30,31], ours is the first report to suggest that NR5A1 regulates DAX-1 expression in the pituitary gonadotropes in vivo. Overall, we identified several candidate NR5A1 downstream genes in the pituitary gonadotrope. Among these, several genes have not yet been linked to pituitary gonadotrope function and should be evaluated in future studies.

## 4. Materials and Methods

### 4.1. Mice

We previously identified a gonadotrope-specific PE of *Nr5a1* [11]. In this study, we deleted the PE region from the mouse genome following a published procedure [32]. Guide RNAs targeting the upstream and downstream regions of the PE were designed using CRISPR direct (http://crispr.dbcls.jp/, accessed on 17 March 2017). crRNA, tracrRNA, and Cas9 protein (Integrated DNA Technologies) were mixed to form an RNP complex and then introduced into the fertilized eggs by electroporation (Genome Editor, BEX). The eggs were then transferred into the oviducts of recipient mothers, and the genotypes of the resulting pups were determined by PCR. The sequences of genotyping primers are shown in Supplemental Table S3. Homozygous PE deletion mice were designated as PE^−/−^ mice, whereas heterozygous PE deletion mice (PE^+/−^ mice) were used as controls unless otherwise noted. Ad4BP-BAC-EGFP mice [33] were used to collect NR5A1-expressing gonadotropes from the pituitary gland via fluorescence-activated cell sorting (FACS).

### 4.2. Tissue Preparation, Histological Analyses, and Immunostaining

Mice were anesthetized with 0.3 mg/kg medetomidine hydrochloride (Nippon Zenyaku Kogyo, Fukushima, Japan), 4 mg/kg midazolam (Astellas Pharma, Tokyo, Japan), and 5 mg/kg butorphanol tartrate (Meiji Seika Pharma, Tokyo, Japan), and then perfused with PBS followed by 4% paraformaldehyde in 0.1 M phosphate buffer (pH 7.4) from the left ventricle. For histological analyses, tissues were embedded in paraffin wax, sectioned to 5 μm in thickness, and subjected to HE or Masson trichrome staining. Stained sections were observed using a BZ-X700 fluorescence microscope (Keyence, Osaka, Japan). For immunostaining, 50-μm thick sections were cut using a cryotome (Leica CM3050 S, Leica Camera AG, Wetzlar, Germany) and stained using the free-floating staining method [13]. The primary and secondary antibodies used in this study are listed in Supplemental Table S4. For nuclear staining, 4′6′-diamidino-2-phenylindole (DAPI) (Sigma-Aldrich, St. Louis, MO, USA) was used. Tissue sections were encapsulated in VECTASHIELD Mounting Medium (Vector Laboratories, Newark, CA, USA) and photographed with a LSM 700 laser scanning microscope (Carl Zeiss AG, Oberkochen, Germany).

### 4.3. RNA Preparation and Quantitative RT-PCR

Total RNA was prepared from the anterior pituitary of PE^+/−^ (n = 3) and PE^−/−^ (n = 3) male mice and subjected to reverse transcription with random hexamers (Superscript VILO master mix, Invitrogen, Carlsbad, CA, USA). Synthesized cDNA was used for quantitative PCR using the AriaMx Real Time PCR system (Agilent, Santa Clara, CA, USA) with gene-specific primers (Supplemental Table S5) and SYBR green qPCR master mix (Agilent, Santa Clara, CA, USA). Expression of the genes of interest was adjusted relative to that of *Actb*, the gene encoding β-actin.

### 4.4. Measurement of Plasma Gonadotropin

Blood samples were collected from the right ventricle of anesthetized PE^+/+^ (n = 6), PE^+/−^ (n = 8), and PE^−/−^ (n = 7) male mice. Plasma LH and FSH concentrations were measured by a double-antibody radioimmunoassay (RIA) with mouse LH- and FSH-RIA kits provided by the National Hormone and Peptide Program (Torrance, CA, USA), as previously described [34,35]. LH and FSH concentrations were expressed in terms of mouse LH-RP (AFP-5306A) and FSH-RP (AFP-5308D), respectively. The lowest detectable level of LH in 25 µL plasma samples was 0.156 ng/mL, and the intra- and inter-assay coefficients of variation were 6.5 and 7.5%, respectively, at 2.8 ng/mL. The lowest detectable level of FSH in 25 µL plasma samples was 1.252 ng/mL, and the intra- and inter-assay coefficients of variation were 8.7 and 8.7%, respectively, at 17.1 ng/mL.

### 4.5. Measurement of Testicular Steroids

Testes were collected from control and PE^−/−^ mice at the fetal stage (E18.5) and adult stage (8–10 weeks after birth), respectively (n = 5 in each experimental condition). Levels of testicular steroids were determined by gas chromatography-mass spectrometry (GC-MS) as previously described [36]. The concentration of steroid hormone was given in units of ng/tissue in the fetal testis, and ng/mg tissue in the adult testis. The metabolic ratio for each enzymatic reaction was calculated by dividing the metabolite concentration by the precursor concentration.

### 4.6. mRNA Sequencing, Data Processing, and Differentially Expressed Gene Analyses

mRNA sequencing analyses were performed as previously described [13]. Briefly, total RNAs were prepared from the whole pituitary gland (control mice and PE^−/−^ mice) or from EGFP-positive cells sorted from Ad4BP-BAC-EGFP mouse pituitary glands, and were then subjected to library construction using NEBNext Single Cell/Low Input RNA Library Prep Kit for Illumina (New England Biolabs, Ipswich, MA, USA). Libraries were subjected to paired-end 150-bp sequencing on an Illumina series sequencer. After removing adapter sequences and low-quality reads using “cutadapt” (version 4.2) with default parameters, FASTQ files were mapped to the mouse genome (mm10) by “STAR” (version 2.5.4a) with default parameters. Reads for each gene were counted using “featureCounts” (version 1.6.1) with default parameters, and gene expression matrix files were subjected to differentially expressed gene analyses using “EdgeR”. Genes with reduced expression in PE^−/−^ mice relative to controls (log_2_FC < −2, *p*-value < 0.05, FDR < 0.05) were extracted. Genes with higher expression in the isolated gonadotropes than in the control whole pituitary were also extracted. We compared the two gene sets, and overlapping genes were then subjected to annotation analyses by “Metascape” [37].

### 4.7. Statistical Analyses

Quantitative RT-PCR data were presented as mean ± SEM, and statistical differences between experimental groups were examined by the two-tailed unpaired Student’s *t*-test. Plasma gonadotropin levels were presented as mean ± SEM, and differences were evaluated by one-way ANOVA followed by Tukey’s post hoc test. Intratesticular steroid levels and metabolic ratios were presented as mean and SD, and comparative levels of testicular steroids and metabolic ratios between control and PE^−/−^ groups were evaluated by a non-parametric Mann–Whitney U test.

## 5. Conclusions

Intronic enhancer plays an essential role in pituitary gonadotrope-specific *Nr5a1* gene expression. NR5A1 regulates functional differentiation of pituitary gonadotropes, and thereby induces development of reproductive organs. This study identified candidate downstream genes of NR5A1 in the pituitary gonadotrope. Some of them have been already shown to be important for the pituitary gonadotrope function. However, we also identified several genes of which function in the pituitary gland is unclear. These genes may be the target of future studies to clarify the pathogenesis of human hypogonadotropic hypogonadism patients.

## Figures and Tables

**Table 1:** Intratesticular steroids in PE^+/−^ mice and PE^−/−^ mice at E18.5.

	Amount (pg/Whole Tissue)				
	PE^+/−^		PE^−/−^		
Steroid Compound	Mean	SD	Mean	SD	*p*-Value ^1^
Dehydroepiandrosterone	28.4	3.1	24.5	8.5	0.5476
Androstenediol	36.7	1.2	39.7	8.7	0.5000
Epitestosterone	12.2	2.1	15.8	9.5	0.6905
5α-androstane-3β, 17β-diol	18.7	11.2	14.9	1.8	0.6905
Androstenedione	324.0	133.2	381.4	156.0	0.5476
Testosterone	627.1	248.0	722.9	274.6	0.6905
Pregnenolone	18.8	4.6	15.5	0.6	0.2222
Progesterone	28.97	0.24	28.68	11.33	0.8413
17α-hydroxyprogesterone	100.3	35.3	98.4	33.6	>0.9999
11-deoxycortisol	49.9	7.9	62.8	16.5	0.1508

^1^ Statical significance was determined using the Mann–Whitney U test.

**Table 2:** Intratesticular steroids in adult PE^+/−^ and PE^−/−^ mice.

	Concentration (pg/mg Tissue)				
	PE^+/−^		PE^−/−^		
Steroid Compound	Mean	SD	Mean	SD	*p*-Value ^1^
3α-androstane-3α, 17β-diol	2.1	0.3	2.3	1.2	0.6905
Dehydroepiandrosterone	0.6	0.1	1.8	0.4	0.0079 *
Androstenediol	3.7	0.8	2.4	0.6	0.0317 *
Epitestosterone	0.9	0.9	1.2	0.8	>0.9999
3α-androstane-3β, 17β-diol	1.1	0.5	1.6	0.5	0.2222
7α-hydroxyandrostenedione	3.1	0.9	12.3	2.7	0.0079 *
Dihydrotestosterone	1.7	0.8	2.3	0.5	0.2222
Androstenedione	54.0	21.7	10.9	5.5	0.0079 *
Testosterone	58.3	57.4	9.7	8.1	0.0079 *
6β-hydroxyandrostenedione	1.3	0.3	ND	ND	NA
6β-hydroxytestosterone	1.5	1.5	ND	ND	NA
Pregnenolon	4.7	1.1	2.8	2.0	0.0952
Progesterone	8.61	3.11	9.95	3.73	0.6905
16α-hydroxytestosterone	5.9	6.8	ND	ND	NA
16α-hydroxyandrostenedione	1.0	0.7	ND	ND	NA
17α-hydroxypregnenolone	1.4	0.3	ND	ND	NA
17α-hydroxyprogesterone	9.6	2.9	7.1	2.9	0.3095
Tetrahydrodeoxycorticosterone	ND	ND	3.9	2.1	NA
Allo-tetrahydrodeoxycorticosterone	0.9	0.4	1.0	0.3	0.5476
11-deoxycortisol	2.9	3.0	3.8	2.5	0.1508
Corticosterone	13.3	12.4	7.7	5.7	0.3095

^1^ Significance was determined using the Mann–Whitney U test. * Statistically significant difference (*p* < 0.05). ND, not detected under the limit of quantification; NA, not applicable because analyte was not detected in either experimental group.

## Data Availability

Sequence data were deposited to the GEO repository with accession numbers GSE216466 and GSE2164685.

## Supplementary Materials

The supporting information can be downloaded at: https://www.mdpi.com/article/10.3390/ijms24010192/s1.
