# Supplementary material for: Intronic Enhancer Is Essential for Nr5a1 Expression in the Pituitary Gonadotrope and for Postnatal Development of Male Reproductive Organs in a Mouse Model
Source: Int J Mol Sci. 2022 Dec 22;24(1):192. doi: 10.3390/ijms24010192 (PMC9820228; doi:10.3390/ijms24010192)
Supplement: Supplementary file 1 [file ijms-24-00192-s001.zip › SupplementaryMaterial_YSrev.docx]

Supplementary Material for

Intronic enhancer is essential for *Nr5a1* expression in the pituitary gonadotrope and for postnatal development of male reproductive organs in a mouse model

**
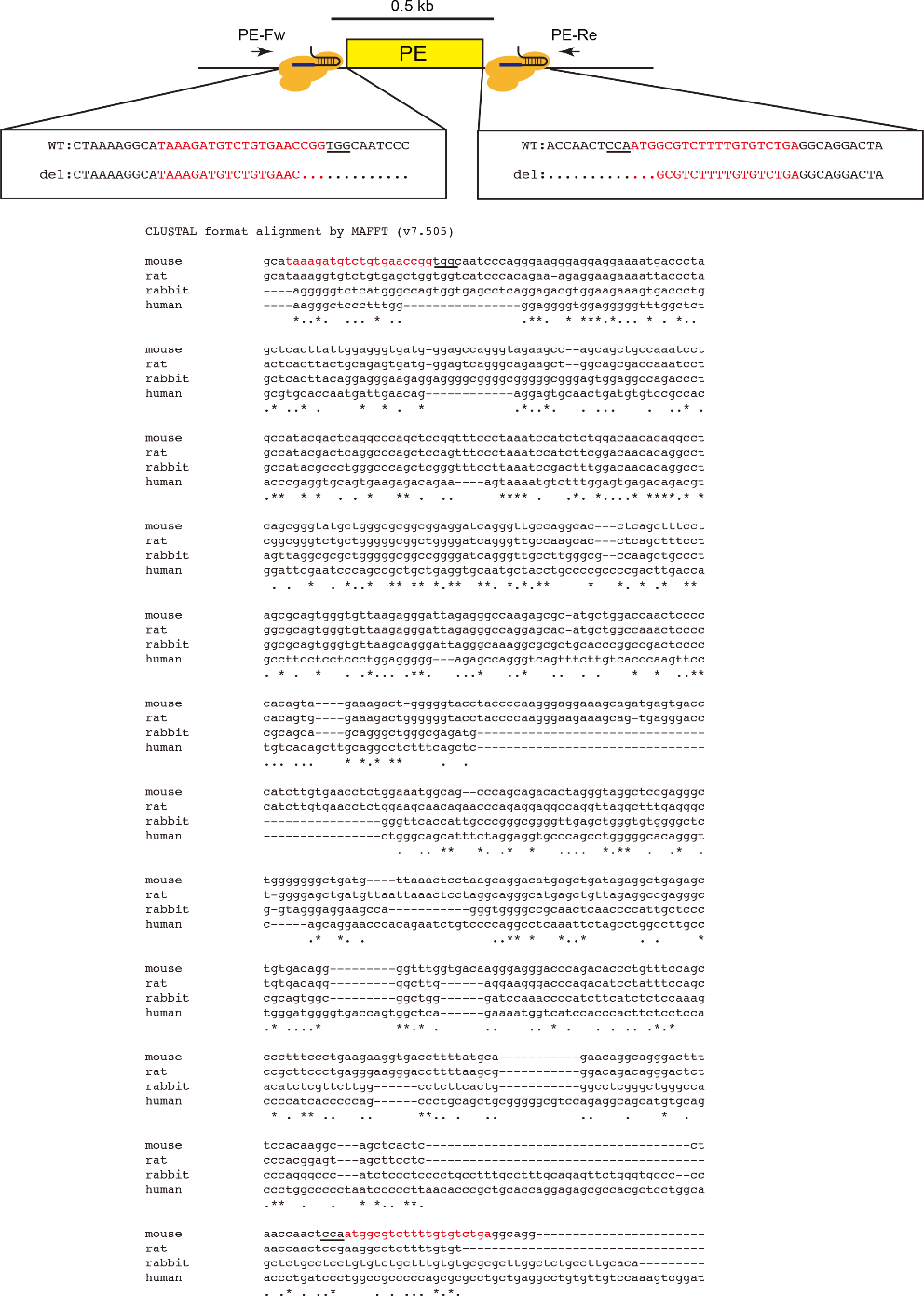
Figure S1**. PE deletion by genome editing. Two guide RNAs were designed to target upstream and downstream regions of the PE. Underlined 3-bp sequences are the protospacer adjacent motif sequences. Nucleotide conservation of PE regions between four mammalian species (mouse, rat, rabbit, and human) is shown. Asterisks indicate the nucleotides conserved in all animal species.

**Figure S2.** Comparison of adrenal glands in control and PE^-/-^ mice. Scale bar: 2 mm


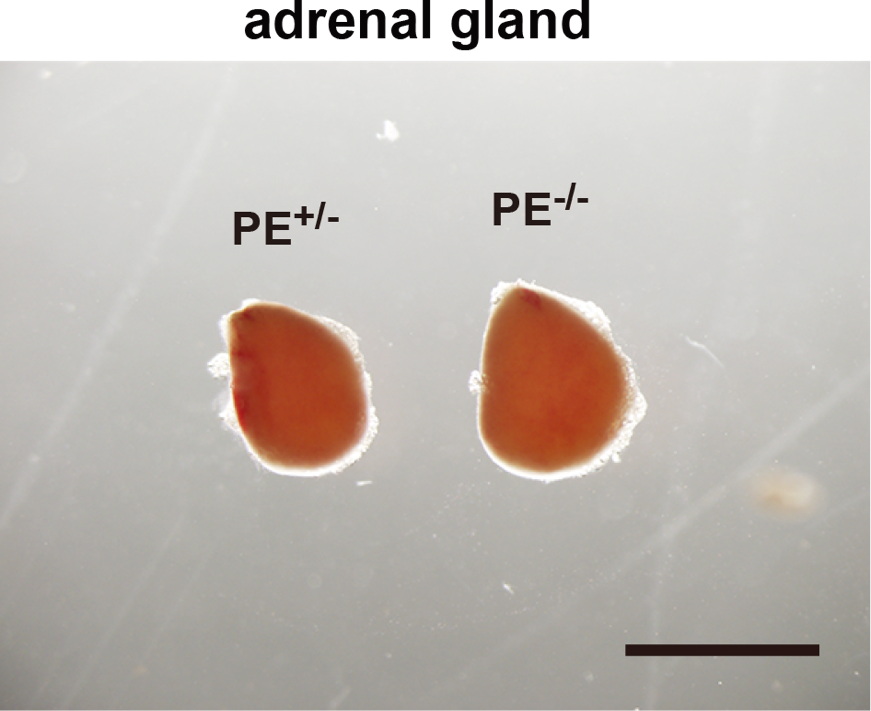


**Figure S3.** Histological changes in the ovaries of PE^-/-^ female mice. (A) Comparison of control and PE^-/-^ mouse ovaries. (B and C) Image of HE-stained ovary sections. (B’ and C’) Magnified view of the areas enclosed by open rectangles in (B) and (C). CL: corpus luteum. Arrows in C’ indicate follicles; arrowheads indicate regressing follicles. Scale bar: 200 µm.


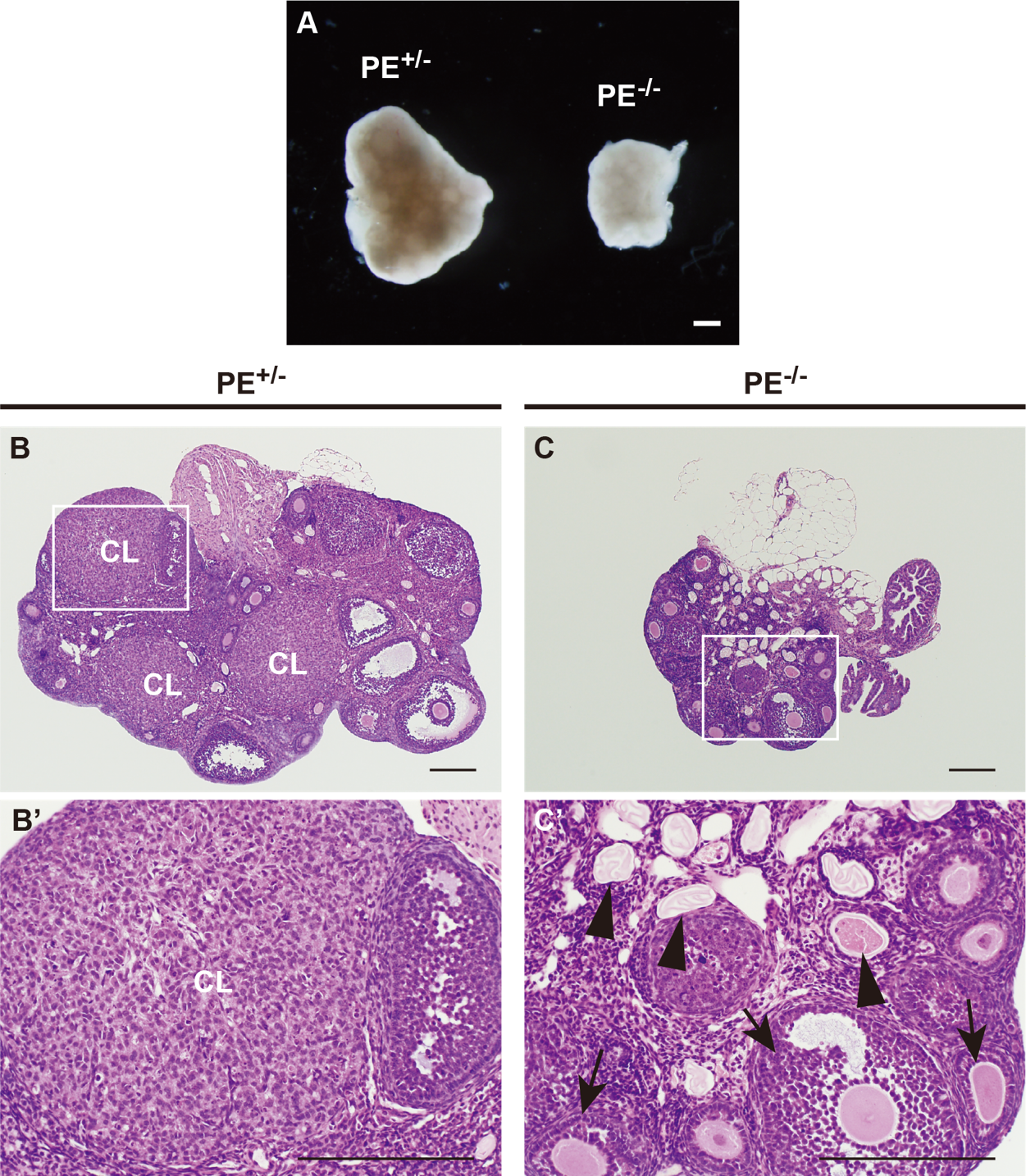


**Figure S4.** Expression of pituitary gonadotrope marker genes in the female pituitary gland. Y-axis indicates expression relative to *Actb* expression. Statistical significance between two experimental groups was evaluated by unpaired *t*-test. *significant difference (p<0.05), NS: not significant.


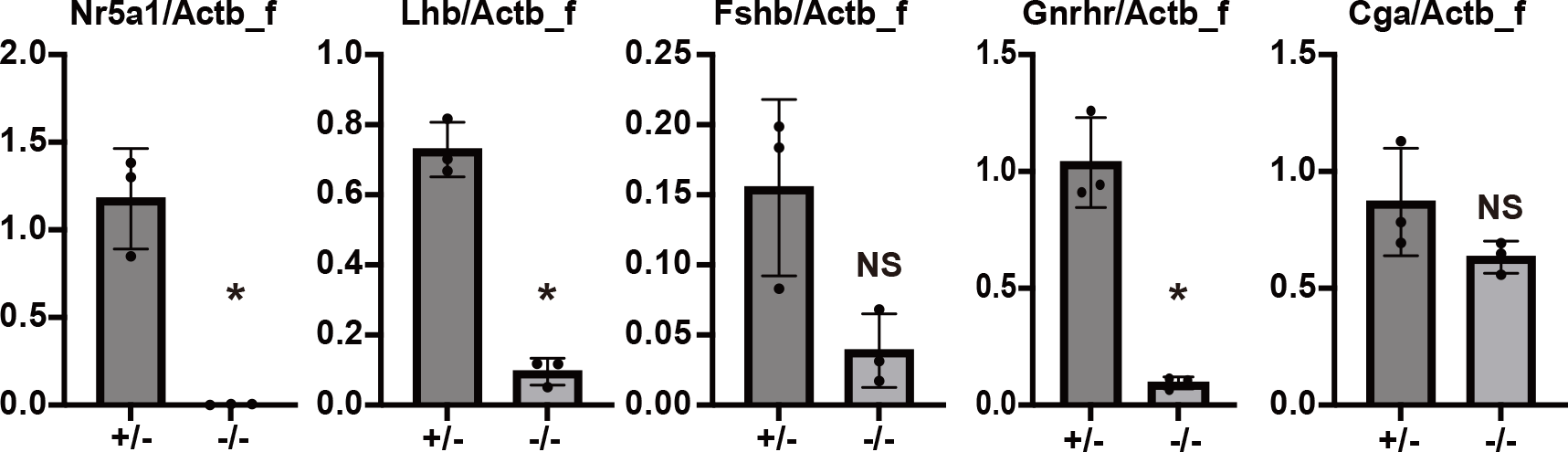


**Figure S5.** Cluster dendrogram of the mRNA sequence data obtained from the whole pituitary gland of a control male mouse (Pit_m_het1-4) and from the gonadotropes isolated from the male pituitary gland (gonadotrope_m1-4).


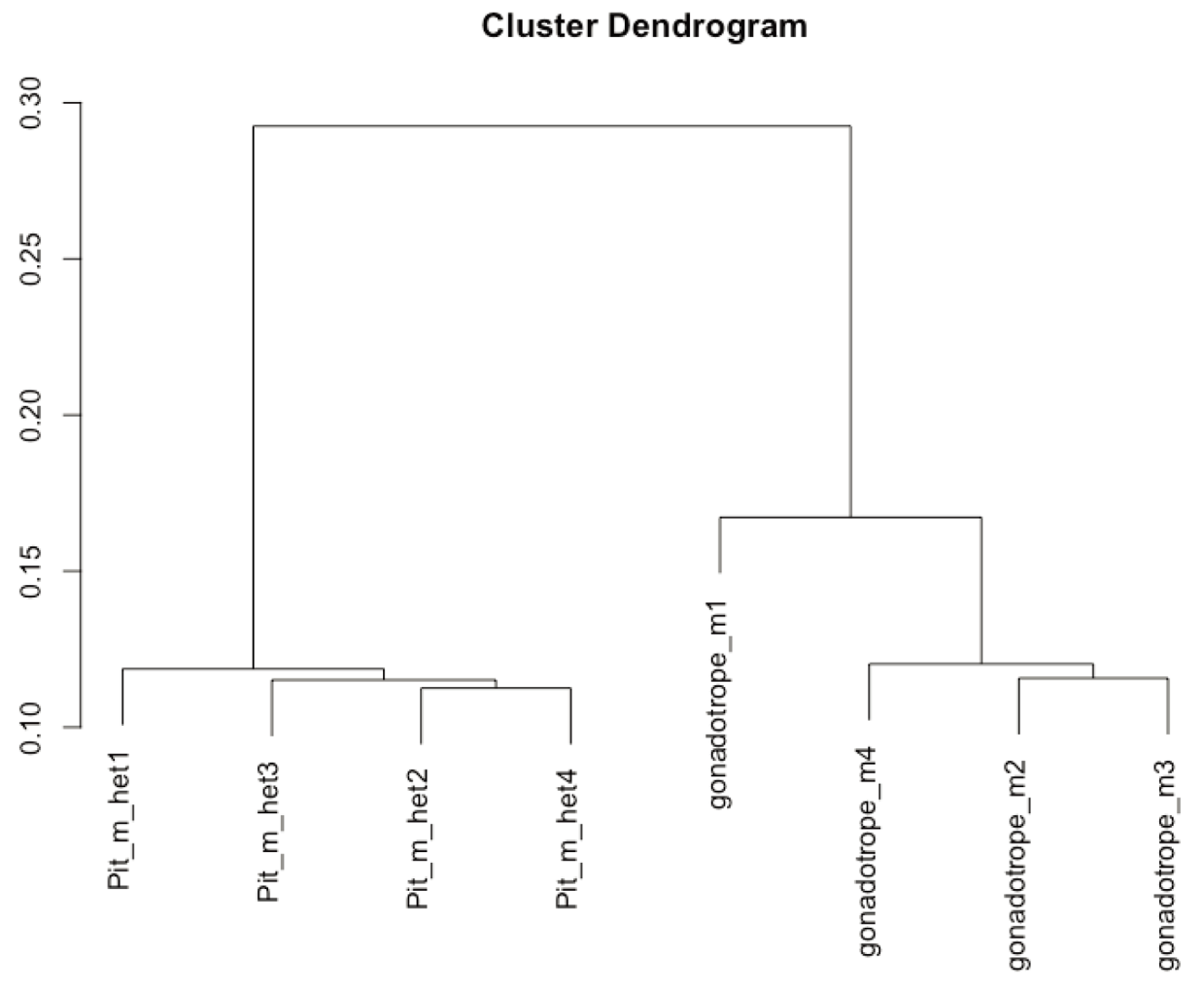


**Figure S6.** Cluster dendrogram of the mRNA sequence data obtained from the pituitary gland of a control female mouse (Pit_f_het1-4) and from the gonadotropes isolated from the female pituitary gland (gonadotrope_f1-4).


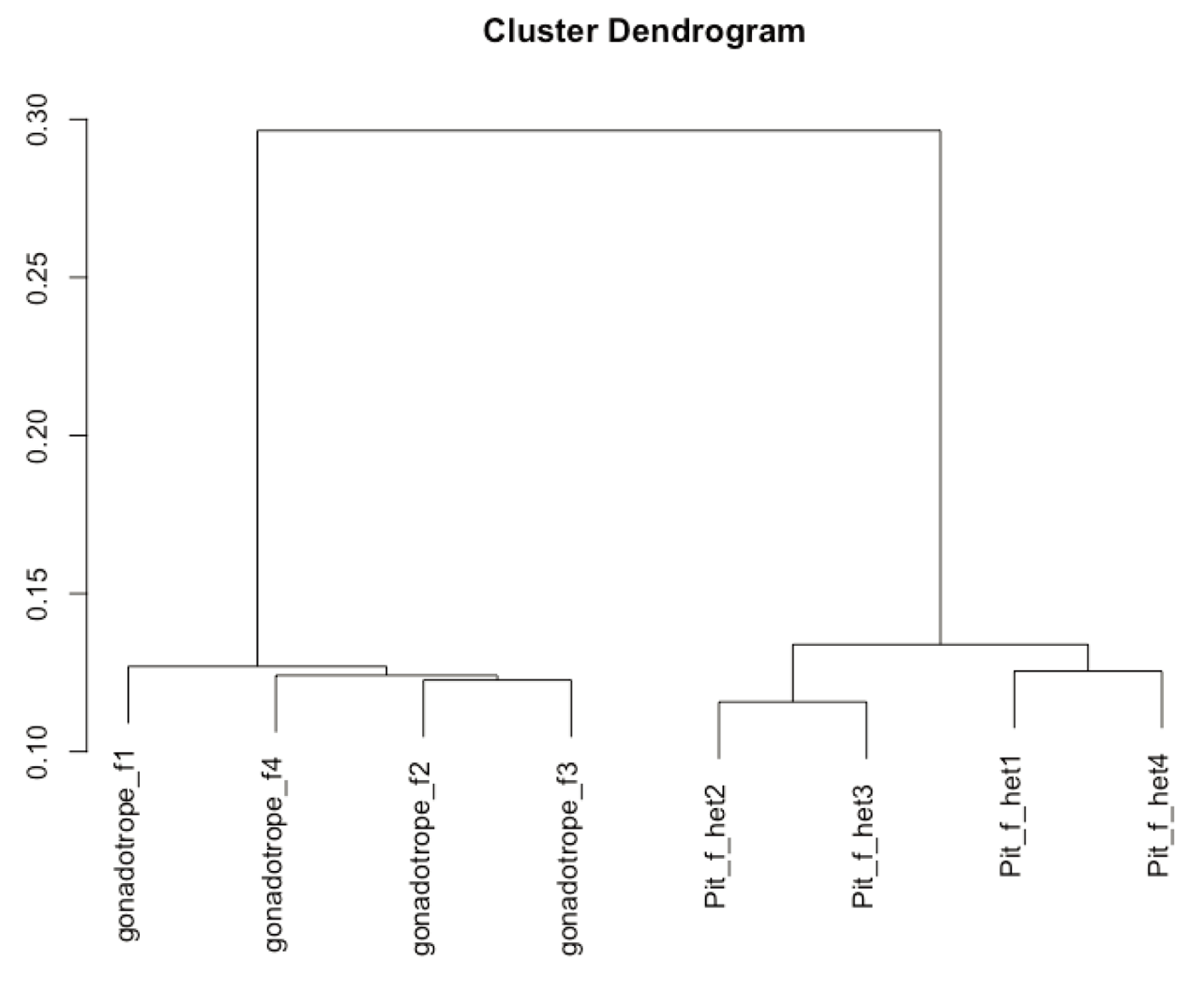


**Figure S7.** Transcriptomic analyses of the whole pituitary and isolated gonadotropes in females. (A) Venn diagram showing an overlap between two gene sets; genes downregulated in the PE^-/-^ pituitary (Pit_f_homo_down) and those with higher expression in the gonadotropes than in the whole pituitary (gonadotrope_f_up). (B) List of the nine overlapping genes between the two gene sets shown in (A), and comparison these genes between the control and PE^-/-^ pituitary. (C) GO terms enriched in the nine genes.


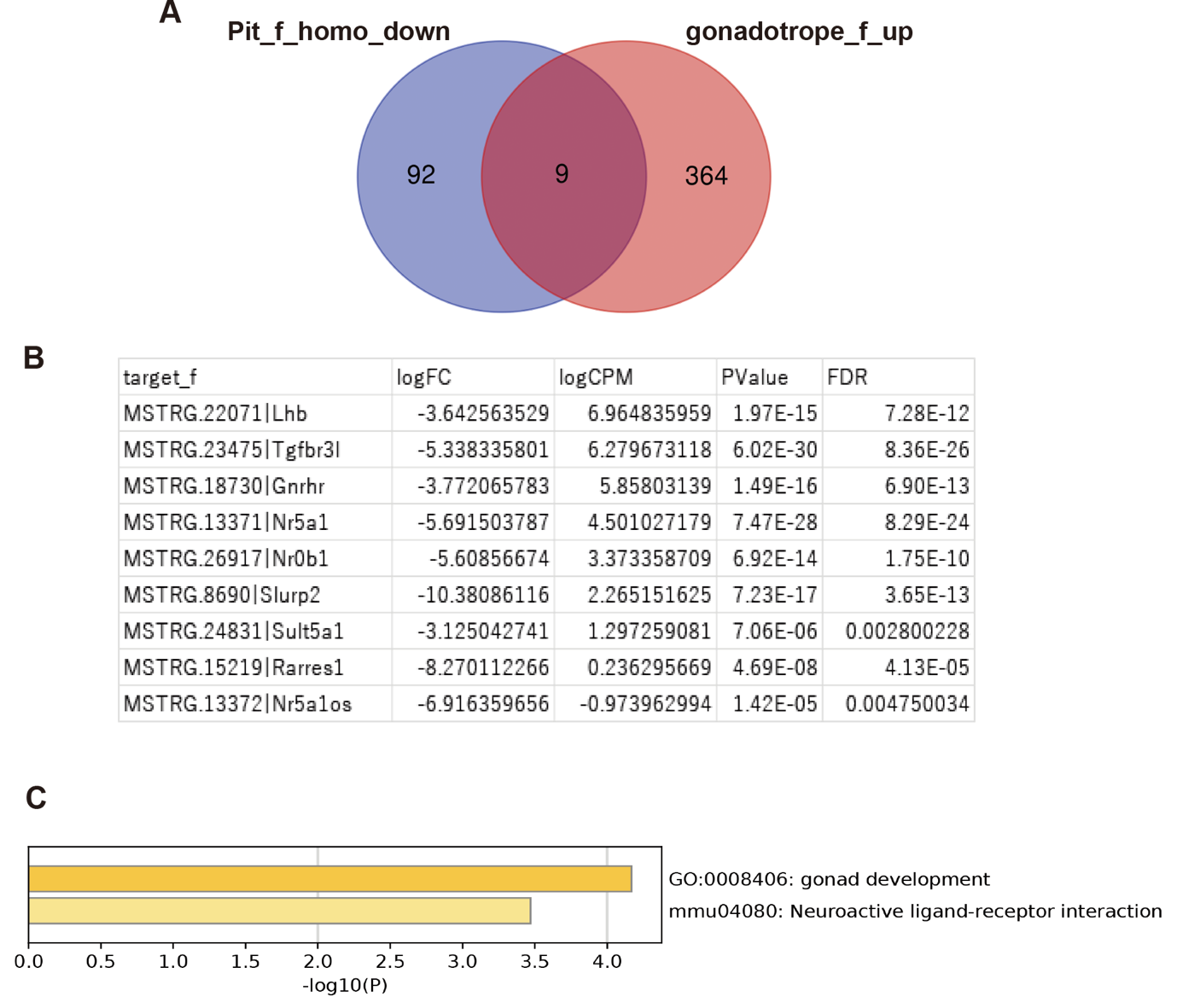


**Table S1.** Metabolic ratios of steroids in PE (+/-) mice and PE (-/-) mice at E18.5.

|  | PE (+/-) | | PE (-/-) | |  |
| --- | --- | --- | --- | --- | --- |
| Metabolic ratio (metabolite/precursor) | Mean | SD | Mean | SD | p-value |
| 17α-hydroxylase (17α-OH-Prog/Prog) | 3.5 | 1.2 | 3.7 | 1.3 | 0.7302 |
| 3β-HSD1 (Prog/Preg) | 1.6 | 0.3 | 1.9 | 0.8 | 0.4206 |
| 21-hydroxylase (11-deoxyF/17α-OH-Prog) | 0.6 | 0.2 | 0.7 | 0.1 | 0.6429 |
| 17,20-lyase (A-dione/17α-OH-Prog) | 3.4 | 1.4 | 4.3 | 2.0 | 0.4206 |
| 3β-HSD1 (A-dione/DHEA) | 11.7 | 5.5 | 17.7 | 9.5 | 0.2222 |
| 3β-HSD2 (T/A-diol) | 17.2 | 7.2 | 18.2 | 5.8 | 0.8413 |
| 17β-HSD1 (A-diol/DHEA) | 1.3 | 0.1 | 1.9 | 1.0 | 0.8413 |
| 17β-HSD1 (T/A-dione) | 2.0 | 0.5 | 2.0 | 0.4 | >0.9999 |
| 17α-HSD1 (epi-T/A-dione) | 0.0 | 0.0 | 0.1 | 0.1 | >0.9999 |

Statical significance was determined using the Mann-Whitney U test.

17α-OH-Prog, 17α-hydroxyprogesterone; Prog, progesterone; Preg, pregnenolone; 11-deoxyF, 11-deoxycortisol, Adione, androstenedione; DHEA, dehydroepiandrosterone; T, testosterone; A-diol, androstenediol; Epi-T, epitestosteron.

**Table S2.** Metabolic ratios of steroids in adult PE (+/-) mice and PE (-/-) mice.

|  | PE (+/-) | | PE (-/-) | |  |
| --- | --- | --- | --- | --- | --- |
| Metabolic ratio (metabolite/precursor) | Mean | SD | Mean | SD | p-value |
| 17α-hydroxylase (17α-OH-Prog/Prog) | 1.2 | 0.6 | 0.7 | 0.3 | 0.1508 |
| 3β-HSD1 (Prog/Preg) | 1.9 | 0.9 | 5.0 | 3.1 | 0.0635 |
| 21-hydroxylase (11-deoxyF/17α-OH-Prog) | 0.3 | 0.2 | 0.5 | 0.2 | *0.0476 |
| 17,20-lyase (A-dione/17α-OH-Prog) | 5.7 | 1.3 | 1.6 | 0.6 | *0.0080 |
| 3β-HSD1 (A-dione/DHEA) | 90.9 | 22.6 | 6.0 | 2.4 | *0.0080 |
| 3β-HSD2 (T/A-diol) | 14.9 | 12.2 | 4.1 | 2.9 | *0.0317 |
| 17β-HSD1 (A-diol/DHEA) | 6.6 | 2.0 | 1.3 | 0.2 | *0.0079 |
| 17β-HSD1 (T/A-dione) | 0.9 | 0.5 | 0.8 | 0.5 | >0.9999 |
| 17α-HSD1 (epi-T/A-dione) | 0.0 | 0.0 | 0.1 | 0.1 | *0.0159 |
| 5α-reductase (DHT/T) | 0.0 | 0.0 | 0.8 | 1.2 | *0.0079 |
| 3α-HSD (αββ-diol/DHT) | 2.4 | 2.5 | 1.6 | 0.8 | 0.8413 |
| 3β-HSD (ααβ-diol/DHT) | 2.5 | 1.0 | 1.0 | 0.1 | *0.0079 |

Statical significance was determined using the Mann-Whitney U test.

*Statistically significant difference (p<0.05)

17α-OH-Prog, 17α-hydroxyprogesterone; Prog, progesterone; Preg, pregnenolone; 11-deoxyF, 11-deoxycortisol, Adione, androstenedione; DHEA, dehydroepiandrosterone; T, testosterone; A-diol, androstenediol; Epi-T, epitestosterone; DHT, dihydrotestosterone; αββ-diol, 3α-androstane-3β,17β-diol ; ααβ-diol, 3α-androstane-3α,17β-diol.

**Table S3.** Primers used for genotyping the PE-deleted mice

| Primer name | Sequence | Amplicon |
| --- | --- | --- |
| PE-Fw | ACAGTTGGGTAGGCTTCACTGT | WT: 889 bp  del: 278 bp |
| PE-Re | CAGACATGCTGTGTGAGAACAA |  |

**Table S4.** Antibodies used for immunostaining assays

Primary antibodies

| Target protein | Source (reference) | Dilution |
| --- | --- | --- |
| NR5A1 | Rat monoclonal  (Shima *et al*., 2008; Yokoyama et al., 2009) | 1:1000 |
| HSD3B  (3β-HSD) | Rabbit polyclonal  (Fatchiyah *et al.*, 2006) | 1:10000 |
| SOX9 | Rabbit polyclonal  (Katoh-Fukui *et al.*, 2011) | 1:10000 |
| LHβ | Rabbit polyclonal  (NHPP, LOT# AFP571292393) | 1:10000 |
| FSHβ | Rabbit polyclonal  (NHPP, LOT# AFPHFSH6) | 1:10000 |
| TSHβ | Rabbit polyclonal  (NHPP, LOT# AFP1274789) | 1:10000 |

Secondary antibodies

| Target protein | Source (reference) | Dilution |
| --- | --- | --- |
| Rat IgG | ALEXA Fluor® 594 goat anti-rat IgG (H+L)  (Thermo Fisher Scientific; A11007) | 1:500 |
| Rabbit IgG | ALEXA Fluor® 488 goat anti-rabbit IgG (H+L)  (Thermo Fisher Scientific; A11008) | 1:500 |

**Table S5.** Primers used for quantitative RT-PCR

| Gene (protein) | Primer direction | Primer sequence (5′ to 3′) |
| --- | --- | --- |
| *Nr5a1*  (Ad4BP/SF-1) | F | AGAGCTGCAAAATCGACAAGAC |
|  | R | CGAATCTGTGCTTTCTTCTGCT |
| *Lhb*  (LHβ) | F | AGTCTGCATCACCTTCACCA |
|  | R | TGAGGGCTACAGGAAAGGAG |
| *Fshb*  (FSHβ) | F | AGAGAAGGAAGAGTGCCGTT |
|  | R | CACAGCCAGGCAATCTTACG |
| *Gnrhr*  (GnRH receptor) | F | TCTATGATCAGCCTGGCCTG |
|  | R | GCTCTGACACCCTGTTCAAC |
| *Cga*  (αGSU) | F | CAAGAAGACAATGCTGGTTCCA |
|  | R | GCACTCCGTATGATTCTCCACT |
| *Actb*  (β-actin) | F | CCATCATGAAGTGTGACGTTGA |
|  | R | ACCGATCCACACAGAGTACTTG |

**Supplementary Matrix File S1.** List of the genes in “Pit_m_homo_down”, “gonadotrope_m_up”, “Pit_f_homo_down” and “gonadotrope_f_up”.
